# Benefits and harm of systemic steroids for short- and long-term use in rhinitis and rhinosinusitis: an EAACI position paper

**DOI:** 10.1186/s13601-019-0303-6

**Published:** 2020-01-03

**Authors:** Valerie Hox, Evelijn Lourijsen, Arnout Jordens, Kristian Aasbjerg, Ioana Agache, Isam Alobid, Claus Bachert, Koen Boussery, Paloma Campo, Wytske Fokkens, Peter Hellings, Claire Hopkins, Ludger Klimek, Mika Mäkelä, Ralph Mösges, Joaquim Mullol, Laura Pujols, Carmen Rondon, Michael Rudenko, Sanna Toppila-Salmi, Glenis Scadding, Sophie Scheire, Peter-Valentin Tomazic, Thibaut Van Zele, Martin Wagenmann, Job F. M. van Boven, Philippe Gevaert

**Affiliations:** 1 Cliniques Universitaires Saint-Luc Brussels, Av. Hippocrate 10, 1200 Brussels, Belgium; 2 Department of Otorhinolaryngology, Amsterdam University Medical Centres, AMC, Amsterdam, The Netherlands; 3 Upper Airway Research Laboratory, Dep. of Otorhinolaryngology, Ghent University Hospital, Ghent, Belgium; 4 Bispebjerg University Hospital, Copenhagen, Denmark; 5 Faculty of Medicine, Transsylvania University, Brasov, Romania; 6 Hospital Clínic, IDIBAPS, CEBERES Universitat de Barcelona, Catalonia, Spain; 7 Centro Medico Teknon, Barcelona, Spain; 8 Department of Ear, Nose and Throat Diseases, Karolinska University Hospital, Stockholm, Sweden; 9 Pharmaceutical Care Unit, Faculty of Pharmaceutical Sciences, Ghent University, Ghent, Belgium; 10 Allergy Unit, Hospital Regional Universitario of Málaga, IBIMA, ARADyAL, Malaga, Spain; 11 Department of Ear, Nose and Throat Disease, University Hospitals, Louvain, Belgium; 12 ENT Department, Guy’s & St Thomas’ Hospital, London, UK; 13 Center of Rhinology and Allergology, Wiesbaden, Germany; 14 Skin and Allergy Hospital, Helsinki University Hospital and University of Helsinki, Helsinki, Finland; 15 University of Cologne, Cologne, Germany; 16 London Allergy and Immunology Center, London, UK; 17 Royal National Throat, Nose and Ear Hospital, London, UK; 18 Medical University Graz, Graz, Austria; 19 Heinrich-Heine-University, Düsseldorf, Germany; 20 Department of Clinical Pharmacy & Pharmacology, University Medical Center Groningen, Groningen Research Institute for Asthma and COPD (GRIAC), University of Groningen, Groningen, The Netherlands

**Keywords:** Glucocorticosteroids, Rhinitis, Rhinosinusitis

## Abstract

Because of the inflammatory mechanisms of most chronic upper airway diseases such as rhinitis and chronic rhinosinusitis, systemic steroids have been used for their treatment for decades. However, it has been very well documented that—potentially severe—side-effects can occur with the accumulation of systemic steroid courses over the years. A consensus document summarizing the benefits of systemic steroids for each upper airway disease type, as well as highlighting the potential harms of this treatment is currently lacking. Therefore, a panel of international experts in the field of Rhinology reviewed the available literature with the aim of providing recommendations for the use of systemic steroids in treating upper airway disease.

## Introduction

Chronic upper airway inflammation is one of the most prevalent chronic disease entities in the world with rhinitis being the most common presentation form affecting 30% of the Western population [1].

Rhinitis is defined as an inflammation of the lining of the nose and is characterized by nasal symptoms including rhinorrhoea, sneezing, nasal blockage and/or itching of the nose. Allergic rhinitis (AR) is the best-known form of non-infectious rhinitis and is associated with an IgE-mediated immune response against allergens [1]. However, a substantial group of rhinitis patients has no known allergy and they form a very heterogeneous non-allergic rhinitis (NAR) patient population suffering from drug-induced rhinitis, occupational rhinitis, irritant-induced rhinitis, hormonally linked rhinitis and idiopathic rhinitis [2, 3]. When inflammation of the nasal mucosa extends to the mucosa of the paranasal sinuses, the consensus term of rhinosinusitis is used. Rhinosinusitis has been shown to affect about 10% of the Western population [4]. In addition to rhinitis symptoms, rhinosinusitis is characterized by postnasal drip, facial pressure and reduction or loss of smell [5]. Acute rhinosinusitis (ARS) is a very common condition and mostly of viral origin [5]. About 0.5–2% of the viral ARS are complicated by a bacterial infection [5].

Chronic rhinosinusitis (CRS) is defined as the presence of two or more nasal symptoms, one of which should be either nasal blockage or nasal discharge, and/or smell problems, and/or facial pain for more than 12 weeks, in combination with inflammatory signs confirmed by nasal endoscopy and/or CT scan. CRS can either present with nasal polyps (CRSwNP) or without (CRSsNP). Additionally, chronic upper airway disease often coexists with lower airway problems, most frequently asthma, but also a link with chronic obstructive pulmonary disease (COPD) and bronchiectasis has been reported [6].

Glucocorticosteroids (GCS) are the oldest and most widely used anti-inflammatory therapy. Since their introduction in the 1950s, GCS have played a key role in the treatment of various inflammatory, allergic, and immunologic disorders. Consequently, they are known as a very effective drug for treating chronic airway inflammatory diseases involving both lower as well as upper airways [1, 4, 7]. GCS can be administered topical or systemically. If possible topical GCS are preferred over systemic GCS treatment as it is well known that this systemic GCS treatment is linked to an extensive range of potential adverse effects (AE’s) that have been well-described in the literature and vary from uncomfortable to life-threatening [8]. Notably, reports on AE and/or toxicity of systemic GCS cover a heterogeneous group of GCS-treated diseases, which complicates the interpretation of the actual risk for the rhinitis/rhinosinusitis patients.

Therefore, the risk–benefit ratio of treating non-life-threatening upper airway diseases with systemic GCS remains debatable and needs clarification.

This document summarizes the current evidence for beneficial as well as harmful effects of administration of systemic GCS in the different types of upper airway disease and aims at providing recommendations about its use in rhinitis and rhinosinusitis based on the current evidence. For each topic 2 experts in the field were appointed to review the literature and topics that were appropriate for clinical recommendations were considered as evidence-based reviews with recommendations. The experts then provided a recommendation based upon the guidelines of the American Academy of Pediatrics (following the recommendation strategy used by the International Consensus on Allergy and Rhinology [9]). Table 1 summarizes the recommendation development based on the combination between levels of evidence and the benefit/harm balance. Generally, the search was focused on adults. Two experts reviewed the literature specifically for the pediatric population.

**Table 1:** American Academy of Pediatrics defined strategy for recommendation development [9]

Evidence quality	Preponderance of benefit over harm	Balance of benefit and harm	Preponderance of harm over benefit
A. Well-designed RCTs	Strong recommendation	Option	Strong recommendation against
B. RCT’s with minor limitations; overwhelming consistent evidence from observational studies	Recommendation		
C. Observational studies (case–control and cohort design)			Recommendation against
D. Expert opinion; case reports; reasoning from first principles	Option	No recommendation	

*RCT* randomized controlled trial

The search was performed in the MEDLINE (Ovid 1946—current; and PubMed 1966—current) and Cochrane databases. The search strategy was based on a combination of MeSH-terms and free text words. Search terms are listed in Additional file 1.

## Mechanisms and actions of GCS

**Fig. 1:**
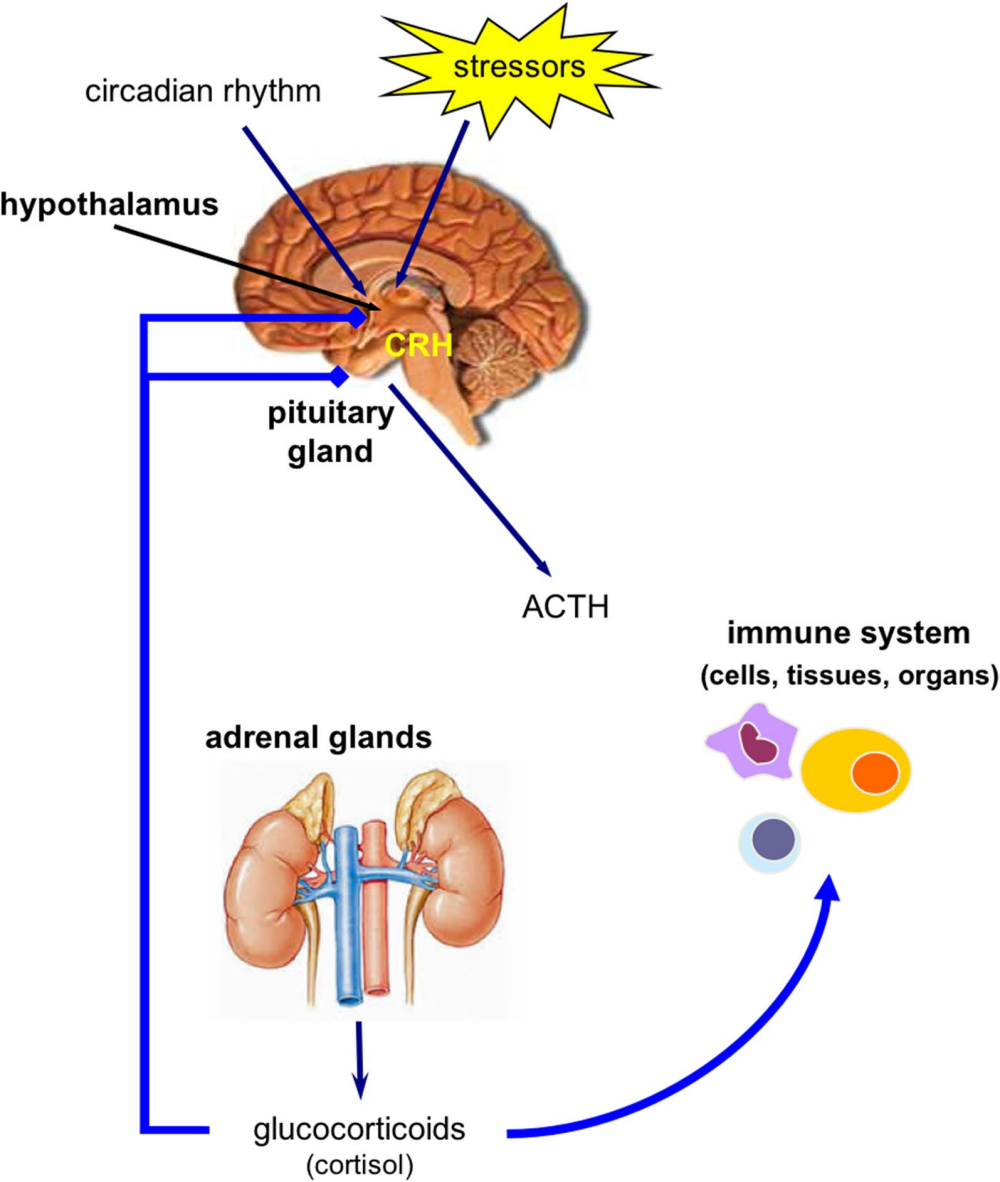
The hypothalamic–pituitary–adrenal axis. Stress stimuli induce the production of CRH by the hypothalamus. CRH induces the production of ACTH by the pituitary gland which stimulates the production of glucocorticoids (cortisol) in the adrenal gland cortex. Cortisol acts on many cells, tissues, and organs including the immune system. The excessive release of cortisol as well as proinflammatory cytokines have a negative feedback on the central nervous system by inhibiting this circadian cycle. *CRH* corticotrophin releasing hormone, *ACTH* adrenocorticotrophin hormone

Corticosteroids, which are produced by the adrenal glands, can be classified as glucocorticoids and mineralocorticoids. Cortisol is the endogenous glucocorticoid in humans, naturally derived from cholesterol metabolism upon stimulation by the hypothalamic–pituitary–adrenal axis (Fig. 1), which is regulated initially by the circadian rhythm, but also by negative feedback by glucocorticoids and glucocorticoid increment induced by stressors such as pain, inflammation or infections [10].

GCS are involved in several physiologic functions. They control the metabolism of carbohydrates, proteins and lipids, as well as the balance of calcium [11, 12]. However, the most explored effects of GCS are the anti-inflammatory and immune-suppressive functions. GCS inhibit the activation and survival of inflammatory cells and modulate the activity of structural cells [13, 14]. The main anti-inflammatory effects of GCS are based on their ability to reduce the synthesis of several cytokines (IL-1, -2, -3, -4, -5, -6, -8, TNF-α, IFN-γ, GM-CSF) from many cells (macrophages, monocytes, lymphocytes, fibroblasts, and epithelial and endothelial cells). This affects recruitment, localization, protein synthesis, and survival of inflammatory cells such as eosinophils [15]. The recruitment of inflammatory cells is also diminished by an inhibited expression of adhesion molecules such as ICAM-1 and VCAM-1 [16], which affects the influx of basophils and mast cells in the epithelial layers of nasal mucosa. Finally, GCS are involved in the pathological wound repair mechanism called remodelling. Remodelled tissue such as the stroma of nasal polyps contains abundant infiltration of inflammatory cells, increased fibroblasts numbers and increased extra-cellular matrix deposition. However, GCS appear to be minimally effective in reversing the structural changes resulting from remodelling [17].

**Fig. 2:**
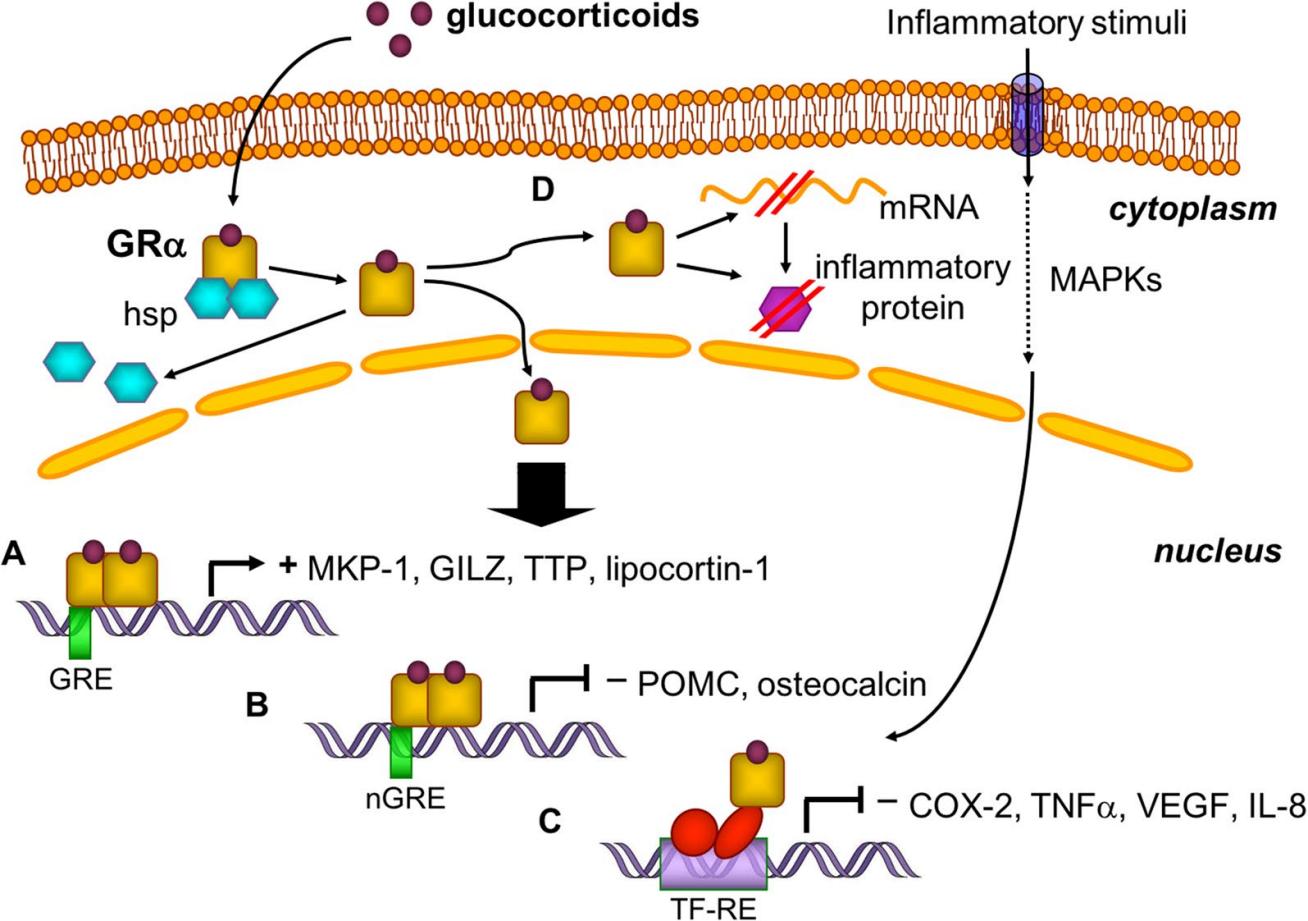
Molecular mechanisms of glucocorticoid action. After crossing the cell membrane by passive diffusion, glucocorticoids bind to GRα, associated heat-shock proteins (HSP) are released, and the ligand bound receptor translocates into the nucleus. Through the activation of MAP kinase (MAPKs) intracellular cascade, inflammatory stimuli induce the production of transcription factors. A GRα dimer can bind glucocorticoid responsive elements (GRE) on the promoter region of target genes and activate anti-inflammatory gene (MKP-1, GILZ, TTP, lipocortin-1) transcription. B Binding of GRα to a negative GRE (nGRE) leads to gene (POMC, osteocalcin) repression. C Protein–protein interactions between GRα and transcription factors (AP-1, NF-κB) repress the transcription of pro-inflammatory genes (COX-2, TNF-α, VEGF, IL-8). D GRα can alter mRNA or protein stability of inflammatory mediators

All these effects are exerted by intracellular activation of the glucocorticoid receptor (GR) [18]. The GR belongs to the superfamily of ligand regulated nuclear receptors [19] and alternative splicing of the GR primary transcript generates two receptor isoforms, named GRα and GRβ. GRα has a widespread distribution in cells and tissues [20], including healthy and diseased upper airway mucosa. Inactive GRα is found primarily in the cytoplasm of cells as part of a large multi-protein complex [21]. Glucocorticoids diffuse across the cell membrane and bind to GRα resulting in a nuclear entry (Fig. 2) [22] where GRβ modulates either positively or negatively the expression of target genes. GRβ has a very low level of expression compared to GRα [20] and acts mainly as a negative inhibitor of GRα-mediated gene modulation [23].

The anti-inflammatory effects of GCS are explained by three broad molecular mechanisms: the decreased expression of pro-inflammatory genes (trans-repression), the increased expression of anti-inflammatory genes (trans-activation), and non-genomic mechanisms. Trans-repression is thought to be mainly due to direct interactions between GRα and pro-inflammatory transcription factors such as the activator protein-1 (AP-1) and NF-κB [24]. Trans-activation is explained by the interaction of GRα to specific target DNA sequences, named glucocorticoid-responsive elements (GRE). Among the genes activated by GRα through GRE with anti-inflammatory functions, there are the mitogen activated protein kinase phosphatase-1, the glucocorticoid inducible leucine zipper and tristetraprolin. In addition, the activated GRα can also reduce inflammation at the post-transcriptional (altering mRNA stability), translational (affecting protein synthesis) and post-translational levels (altering protein processing, modification or degradation) (Fig. 2). For example, the expression of cyclooxygenase-2, TNF-α and GM-CSF are regulated by one or more of these postgenomic mechanisms [25].

Increased expression of GRβ has been reported in different inflammatory diseases, including asthma, and nasal polyposis and has been proposed as one of the potential mechanisms explaining GC resistance [26]. The expression of GRβ is higher in nasal polyps than in nasal mucosa epithelial cells and correlates with increased infiltration of inflammatory cells [27]. Although down-regulation of GRα after treatment with glucocorticoids has been reported [28] and could account for secondary steroid resistance, a recent study in patients in patients with nasal polyps has shown that this effect does not occur in vivo [29].

## Evidence for efficacy of systemic GCS in different inflammatory upper airway diseases

### 1. Allergic rhinitis

AR is the most prevalent presentation form of all allergic diseases and the most com-mon chronic disorder in children. It is considered a risk factor for the development of asthma and a major public health problem, due to its prevalence and impact on patients’ quality of life, work/school performance, and economic burden [30].

Intranasal GCS and oral/topical antihistamines are the most effective symptomatic treatment for AR and should be the first-line therapy for mild to moderate disease [30, 31]. Moderate to severe disease not responsive to intranasal GCS, should be treated with additional pharmacological therapies (including cromolyns and leukotriene receptor antagonists), allergen immunotherapy (AIT) and non-pharmacologic therapies (such as nasal irrigation) [30, 31]. Usually a combination of intranasal GCS and a topical or oral antihistamine is used for moderate to severe AR.

Regarding the use of systemic GCS in AR, the current evidence is scarce. Three studies compared the effect of systemic GCS in adult patients (> 15 year old) with AR (Table 2).

**Table 2:** Summary of the evidence for ‘efficacy of systemic steroids in AR in adults’

Study	Year	LOE (1a to 5)	Study design	Study groups	Clinical end-point efficacy	Conclusion
Borum et al.	1987	1b	RCT	1. 80 mg MP (n = 12 adults with AR) vs. placebo early in the season (n = 12 adults with AR) 2. 80 mg MP (n = 12) vs. placebo late in the season (n = 12)	1. Nasal and ocular symptoms 2. Number of sneezings and nose blowing/day	The effect of MP on nasal blockage is marked and last for 4 weeks MP administration before the pollen season is effective but not recommended in clinical practice to avoid too widespread use
Laursen et al.	1987	1b	RCT	1. 10 mg betamethasone dipropionate IM single dose and oral placebo (n = 17 adults with AR) × 3 weeks 2. 7.5 mg oral prednisolone × 3 weeks and IM placebo (n = 19 adults with AR)	1. Nasal and ocular symptoms 2. Blood eosinophils	Both treatments equally controlled hay fever symptoms Reduction of blood eosinophils with both drugs
Brooks et al.	1993	1b	RCT	1. Placebo (n = 7 adults with AR) 2. 6 mg MP (n = 8 adults with AR) 3. 12 mg MP (n = 8 adults with AR) 4. 24 mg MP (n = 8 adults with AR)	1. Nasal and ocular symptoms 2. Dose–response effect 3. Minimal effective dose 4. Relative effectiveness against various symptoms	MP produced dose-related reduction in all symptoms 24 mg MP reduced significantly all symptoms except nasal itching 6 mg MP reduced significantly nasal congestion, drainage, and eye symptoms Not all rhinitis symptoms responded equally to corticoid treatment. Those that responded least could reflect histamine effect, which was not effectively suppressed by low-dose, short-term corticoid treatment

*RCT* randomized controlled trial, *MP* methylprednisolone, *AR* allergic rhinitis, *IM* intramuscular

The first randomized controlled trial (RCT) from 1987 showed a beneficial effect of a depot injection of 80 mg methylprednisolone (MP) vs. placebo on nasal obstruction and eye symptoms in 48 AR patients, which lasted for 4 weeks [32]. The second study by Brooks et al. [33] investigated the efficacy of different doses of oral MP and placebo in patients not treated with other medications. Thirty-one patients were randomized to receive 0, 6, 12, or 24 mg MP. Oral GCS produced dose-related reduction in all symptoms. The difference between placebo and 24 mg MP was significant for all the symptoms monitored, except itching, which benefited marginally. With 6 mg MP, congestion, drainage, and eye symptoms showed significant drug-placebo differences, but itching, running/blowing, and sneezing did not. The third study by Laursen et al. [34] compared prednisone 7.5 mg for 3 weeks with a single intramuscular injection of betamethasone dipropionate also in patients not treated with other medications. This study showed a therapeutic index in favour of the depot injection versus oral treatment in AR [33].

Despite the therapeutic benefits of systemic GCS in the treatment of AR that were shown in these studies, their use is strongly recommended against in view of the AE’s GCS that are discussed below, and a short course of systemic GCS is only indicated in rare cases. These cases include patients with severe symptoms who do not respond to other drugs, or those who are intolerant to intranasal drugs [1, 35]. Systemic GCS should never be considered as a first-line of treatment for AR [1]. Consequently, oral GCS can be used for a few days as in carefully selected cases when other medical treatment options have failed.

- Evidence level: B.
- Benefits–harm assessment: AE’s of systemic GCS outweigh advantages of therapeutic value, except for patients suffering from very severe and therapy-resistant symptoms.
- Recommendation: Strong recommendation against. Option in patients suffering from very severe and therapy-resistant symptoms.

### 2. Non-allergic rhinitis

Although, the prevalence of NAR among the chronic rhinitis patients ranges from 20 to 50% [36], their disease mechanisms and treatment options are much less studied than their allergic peers. NAR comprises a heterogeneous group of chronic rhinitis subtypes, such as drug-induced rhinitis, hormonal-induced rhinitis, some forms of occupational rhinitis and rhinitis linked to systemic diseases [37]. However, in about 50% of the NAR patients, no specific causal factor can be found and this is addressed as idiopathic rhinitis (IR) [37]. Up till now, no studies are available that investigate the effectiveness of systemic steroids in NAR or IR patients. However, since it is believed that in IR neurogenic pathways are involved, rather than classical inflammatory pathways [38], systemic GCS are not the therapy of choice. Of note, all IR patients included in a recent study investigating the effect of capsaicin in IR, reported lack of clinical response to intranasal GCS [38]. By extrapolation, there is a low likelihood of oral GCS being effective in this patient population, unless more than one etiologic or inflammatory mechanism underlies the development of rhinitis.

Only in selected cases of other subtypes of NAR, such as rhinitis linked to vasculitic or systemic diseases, oral GCS might play a role in the treatment strategy (see below) [39]. Although oral GCS are often prescribed in patients suffering from rhinitis medicamentosa to overcome the withdrawal period of topical decongestants, there are no valuable studies supporting this clinical practice.

- Evidence level: D.
- Benefits–harm assessment: AE’s of systemic GCS outweigh advantages of therapeutic value.
- Recommendation: Recommendation against.

### 3. Acute rhinosinusitis

Compared to the literature on effectiveness of systemic GCS in CRS, data on acute rhinosinusitis (ARS) are scarce. In 2014 an update of a Cochrane review was published [40] concluding that systemic GCS as a monotherapy are ineffective compared to placebo in ARS patients, but might have a beneficial effect on short-term symptom relief when used as an adjunctive therapy to antibiotics.

Up to date, five randomized, placebo-controlled trials investigating the effect of oral GCS in adults with ARS are available and included in the Cochrane meta-analysis (Table 3). From those, only one focused on systemic GCS as a monotherapy [41]. In this high-quality second-line clinical trial, patients with clinically diagnosed ARS were randomized to receive either prednisolone 30 mg/day or placebo for 7 days. In the 174 patients who completed the trial, no clinically relevant benefit of prednisolone over placebo was found regarding facial pain or pressure, other nasal symptoms or quality of life.

**Table 3:** Summary of the evidence for ‘efficacy of systemic steroids in acute rhinosinusitis in adults’

Study	Year	LOE (1a to 5)	Study design	Study groups	Clinical end-point efficacy	Conclusion
Cannoni et al.	1990	1b	RCT	1. Adults with (sub)acute, non-allergic sinusitis with antibiotics and prednisolone 40–60 mg/day for 7 days 2. Adults with (sub)acute, non-allergic sinusitis with antibiotics and NSAID for 7 days	1. Therapeutic success defined as combination of resolution of pain and absence of nasal discharge clinically and endoscopically at day 7	Beneficial effect of prednisolone in combination with antibiotics
Gehanno et al.	2000	1b	RCT	1. Adults with acute sinusitis treated with antibiotics and methylprednisolone 8 mg; 3×/day for 5 day 2. Adults with acute sinusitis treated with antibiotics and placebo for 5 days	1. Clinical recovery on d 14 2. Course of symptoms on day 4 3. Symptoms and radiological signs on day 30	Significant pain relief in combination with antibiotics compared to antibiotics in monotherapy, no additional effect on nasal discharge
Klossek et al.	2004	1b	RCT	1. Adults with acute maxillary sinusitis treated with antibiotics and prednisone 0.8–1.2 mg/kg/day for 3 days 2. Adults with acute maxillary sinusitis treated with antibiotics and placebo for 3 days	1. Difference in VAS for pain at baseline and day 3 2. Differences in VAS for nasal obstruction 3. Time to pain relief 4. Administration of paracetamol 5. Global subjective effect of treatment on day 3 6. Global subjective effect of treatment on days 10–12	Benefit of short course treatment of prednisone in combination with antibiotics vs. antibiotics with placebo
Ratau et al.	2004	1b	RCT	1. Adults with acute sinusitis treated with antibiotics and betamethasone 1 mg/day for 5 days 2. Adults with acute sinusitis treated with antibiotics and placebo for 5 days	1. Improvement of symptoms between day 0 and day 6 2. Percentage of participants with physical signs present or absent on day 0 and day 6 3. Number of paracetamol tablets taken	Benefit of steroids treatment in combination with antibiotics vs. antibiotics with placebo
Venekamp et al.	2012	1b	RCT	1. Adults with acute sinusitis treated with prednisolone 30 mg/day for 7 days 2. Adults with acute sinusitis treated with placebo for 7 days	1. Resolution of facial pain/pressure at day 7 2. Resolution of other clinically relevant symptoms on day 7 3. Time to resolution of total symptoms 4. Median duration of symptoms 5. Quality of life 6. Resumption of daily activities	No clinically beneficial effects of systemic corticosteroid monotherapy

*RCT* randomized controlled trial, *MP* methylprednisolone, *NSAID* non-steroidal anti-inflammatory drug

Four other RCTs investigated the adjunctive effect of systemic GCS to oral antibiotics in ARS. Gehanno et al. [42] reported the adjuvant effect of 5 days of 3 × 8 mg MP/day to amoxicillin–clavulanate in 417 patients. On day four, patients showed significantly less pain in the steroid group whereas nasal discharge did not significantly improve. The use of additional medication was not reported.

In 2004, two similar studies were published; a French study [43] showed a beneficial effect on pain with oral prednisone as an add-on therapy to cefpodoxime in 291 ARS patients. Also Ratau et al. [44] reported a significant benefit of 1 mg of oral betamethasone per day as adjunct to amoxicillin–clavulanate in 42 patients.

In 1990 Cannoni already published similar findings showing a better symptom resolution in ARS patients treated with 40 mg prednisolone/day in combination with antibiotics, compared to patients receiving a non-steroidal anti-inflammatory drug (NSAID) with antibiotics [45].

Altogether, these limited data suggest that systemic GCS as a monotherapy appear to be ineffective in ARS patients. However, oral GCS in combination with antibiotics may be modestly beneficial for short-time symptom relief in adults suffering from ARS, compared to antibiotics alone, with a number needed to treat of seven [40]. Due to the small number of included studies (n = 5) and their methodological bias, a definite conclusion would only be justified if large controlled trials would be available. Given the self-limiting nature of ARS, the relatively small additional clinical benefit of adding GCS to antibiotics, and the potential AE’s, GCS should not be used routinely, but may be considered an option after informed discussion and shared decision making with the patient in the setting of severe pain.

- Evidence level: B.
- Benefits–harm assessment: AE’s of systemic GCS outweigh advantages of therapeutic value in mild and moderate disease.
- Recommendation: Strong recommendation against when only mild to moderate symptoms. Option in patients suffering from severe headaches/symptoms when combined with antibiotics.

### 4. Chronic rhinosinusitis without nasal polyps

For clinical purposes, the definition of CRS includes nasal polyposis (NP) and currently it is still unclear why some CRS patients develop NP and others do not. CRSsNP is characterized by basement membrane thickening, goblet cell hyperplasia, fibrosis, subepithelial oedema and influx of inflammatory cells that are mainly of the neutrophilic subtype with a cytokine pattern deviated towards the Th1 subtype [5].

Based on available data, medical therapy for CRS should begin with daily application of intranasal steroids in conjunction with saline irrigation and subsequent therapies are based on the patient’s severity of symptoms and/or quality of life impairment [4].

There is limited data showing efficacy of oral GCS in CRSsNP and a systematic review analysed the available literature in 2011 [46].

No RCT investigated the effects of oral GCS in CRSsNP and only two retrospective case series in adults are available [47, 48] that both considered CRSwNP and CRSsNP patients, but sub-group analysis allowed an evaluation specific to CRSsNP (Table 4). Both retrospective studies investigated the effects of oral prednisone in conjunction with 1 month of oral antibiotics added to intranasal steroids and irrigations. Improved subjective and objective outcomes were seen after multimodality treatment schemes in both studies for CRSsNP. The study of Subramamian et al. [48] pooled both CRSwNP and CRSsNP patients and found that the CRSsNP patients had better outcomes than CRSwNP patients. Lal et al. [47] demonstrated that the CRSsNP patients showed total symptom resolution 2 months after treatment of 54.9% compared to 51% for the total CRS group. There are no studies available that investigated the benefits of systemic GCS in monotherapy in treating CRSsNP.

**Table 4:** Summary of the evidence for ‘efficacy of systemic steroids in CRSsNP in adults’

Study	Year	LOE (1a to 5)	Study design	Study groups	Clinical end-point efficacy	Conclusion
Subramanian et al.	2002	4	Retrospective	CRS patients (23 CRSsNP and 17 CRSwNP) treated with 1 month antibiotics + intranasal steroids + prednisone tapered over 10 days (20 mg 2×/day for 5 days, 20 mg 1×/day for 5 days). Mostly adult patients (2 patients under 18)	Change in CT scores, symptom scores post-treatment. Time to relapse	Beneficial effect of multimodal therapy on scoring of CT, symptoms or both in 90% of all CRS patients, no specific subanalysis for CRSsNP. Beneficial effect continued beyond 8 weeks in 60% of patients. No subanalysis made for CRSsNP
Lal et al.	2009	4	Retrospective	Adult CRS patients (23 CRSsNP and 17 CRSwNP) treated with antibiotics + intranasal steroids + intranasal decongestants + prednisone tapered over 12 days (60, 40, 20, 10 mg for 3 days each)	Complete endoscopic and symptomatic resolution of symptoms 2 months after start of treatment	Beneficial effect of treatment in 54.9% of CRSsNP

*CRS* chronic rhinosinusitis, *CRSsNP* chronic rhinosinusitis without nasal polyps, *CRSwNP* chronic rhinosinusitis with nasal polyps

Because of a lack of RCTs or even prospective studies, evidence for clinical efficacy of oral GCS therapy in CRSsNP is Level 4 or 5 and in view of the AE discussed later on, not recommended for the management of CRSsNP.

- Evidence level: C.
- Benefits–harm assessment: AE’s of systemic GCS outweigh advantages of therapeutic value.
- Recommendation: Recommendation against.

### 5. Chronic rhinosinusitis with nasal polyps

CRSwNP is different from CRSsNP by the presence of nasal polyps consisting of a large quantity of extracellular oedema with the presence of a dense inflammatory cell infiltrate [49, 50], which is characterized in about 80% of the Caucasian CRSwNP patients, by activated eosinophils [51, 52] and is associated with a predominant Th2 cytokine profile (IL-4, IL-5, IL-10, eotaxin) [53, 54].

A recent suite of Cochrane Reviews has considered the efficacy of interventions for CRSwNP. Two reviews were performed with respect to short-term oral GCS; one comparing oral GCS alone versus placebo or other treatment [55], and a second comparing oral GCS used as an adjunct to other treatments, versus control [56].

For oral GCS alone, 8 trials with a total of 474 participants, all of whom were adult patients CRSwNP, were identified [57–64]. All studies followed up patients to the end of the treatment course, and 3 followed patients for 3 to 6 months after completion. Patients receiving oral GCS achieved better quality of life (standardized mean difference (SMD) of − 1.24 95% CI − 1.92 to − 0.56, measured with RSOM-31), lower nasal symptom scores (SMD − 2.84, 95% CI − 4.09 to − 1.59) and greater polyp reduction (SMD − 1.21) than control groups at the end of the course of treatment. However, there was no difference between groups at 3 to 6 months after the course of treatment.

Treatment doses utilized in included studies included prednisone at 30 mg and reduced over 14 days, prednisolone at 60 mg reducing over 17 days, or at constant dosage of 50 mg or 25 mg for 14 days, or reducing dosages of MP over 20 days. Of the three studies that followed patients beyond the course of treatment, 2 prescribed ongoing intranasal GCS after completion of the systemic dose to both groups while one did not [58, 62, 63].

Included trials were considered to be at low risk of bias, but overall the quality of evidence was rated as low due to the small numbers of participants, heterogeneity of outcome measures and limited follow-up time in most studies.

Another trial considered oral GCS versus placebo as an adjunct to treatment with intranasal GCS in CRSwNP patients [65]. This study recruited 30 participants and was considered at high risk of bias because of lack of blinding and lack of information on randomization. It reported greater reduction in polyp size in the active treatment arm (MD − 0.46, 95% CI − 0.87 to − 0.05).

One trial included in the Cochrane review of oral GCS as an adjunctive treatment recruited children [66] and is therefore considered later in this document.

Table 5 summarizes the evidence of these studies and provides a recommendation for the treatment of CRSwNP by systemic GCS. There is good evidence that systemic GCS are effective in the management of CRSwNP, at least in the short-term. However, considering the evolving understanding of CRSwNP and the chronicity of this condition, the short-lived benefits of systemic GCS therapy need to be balanced with the long-term potential AE’s which are discussed below. Therefore, systemic GCS should not be considered as a first line of treatment for CRSwNP. They can be used in a short course during 2–3 weeks as a last resort of treatment when combinations of other medications are ineffective.

**Table 5:** Summary of the evidence for ‘efficacy of systemic steroids in chronic rhinosinusitis with nasal polyps’

Study	Year	LOE (1a to 5)	Study design	Study groups	Clinical end-point efficacy	Conclusion
Alobid et al.	2014	1b	Prospective non-blinded RCT	Adult CRSwNP patients treated with intranasal budesonide 800 μg daily for 2 weeks in combination with oral prednisone (30 mg daily for 4 days followed by a 2-day reduction of 5 mg) (n = 67) or nothing (n = 22)	1. Polyp grade measured by CT 2. Nasal congestion 3. Loss of sense of smell 3. Polyp tissue eosinophils 4. Nasal nitric oxide	Combined oral and intranasal corticosteroids improve smell and nasal congestion, decrease tissue eosinophilia and increased detection of nNO
Benitez et al.	2006	1b	Prospective non-blinded RCT	Adult CRSwNP patients treated with oral prednisone, 30 mg for 4 days and a 2-day reduction of 5 mg for a total duration 14 days followed by intranasal budesonide for 12 weeks (n = 63) or no treatment (n = 21)	1. Disease individual symptom scoring of nasal obstruction, loss of sense of smell, rhinorrhoea and sneezing 2. Polyp size measured by endoscopy 3. Nasal flow measurements	14 days of oral steroids improved all nasal symptoms, polyp size, and nasal flow, which is maintained by intranasal steroid
Ecevit et al.	2015	1b	Prospective double-blind RCT	Adult CRSwNP patients treated with oral prednisolone, 60 mg/day (6 tablets per day) for 7 days, then reduced to 10 mg (1 tablet) taken every other day, stopping on day 17 (n = 11) or placebo (n = 10)	1. Visual analogue scale for sense of smell, nasal discharge, nasal obstruction and pressure over the sinuses 2. Smell testing 3. Peak nasal inspiratory peak flow	The improvement in the corticosteroid group in the VAS scores, smell tests and PNIF values showed statistically significant differences compared to the placebo group
Hissaria et al.	2006	1b	Prospective double-blind RCT	Adult CRSwNP patiets treated with prednisolone, 50 mg/day for 14 days (n = 20) or placebo (n = 21)	1. Health-related quality of life (RSOM-31) 2. Physician assessment of nasal symptoms (congestion, hyposmia, rhinorrhoea, sneezing, postnasal drip and itch) 3. Polyps size measured by endoscopy 4.MRI of the paranasal sinuses	The prednisolone-treated group showed significant improvement in nasal symptoms. The RSOM-31 improved in both groups, but the prednisolone-treated group had significantly greater improvement than the placebo group. There was significant reduction in polyp size, as noted with nasendoscopy (P < 0.001) and MRI (P < 0.001), only in the prednisolone-treated group
Kapucu et al.	2012	2b	Prospective unblinded RCT	Adult CRSwNP patients treated with oral methylprednisolone 1 mg/kg/day. The dose was applied for 3 days and tapered gradually, with a reduction rate of 8 mg/3 days (n = 12) or no medication (n = 12)	Apoptotic index	Statistically significant differences in apoptotic index were found between each steroid-medicated group and the control group
Kirtsreesakul et al.	2012	1b	Prospective double-blind RCT	Adult CRSwNP patients treated with oral prednisolone 50 mg daily for 14 days (n = 67) or placebo (n = 47)	1. Symptom scoring for blocked nose, runny nose, sneezing, nasal itching, hyposmia, postnasal drip, cough and sinonasal pain 2. Nasal polyp size measured by endoscopy	The prednisolone-treated group showed significantly greater improvements in all nasal symptoms, nasal flow and polyp size than the placebo-treated group
Vaidyanathan et al.	2011	1b	Prospective double-blind RCT	Adult CRSwNP patients treated with prednisolone tablets, 25 mg/day, 2 weeks (n = 30) or placebo (n = 30) in patients on intranasal steroids	1. Juniper mini rhinoconjunctivitis Quality of Life Questionnaire 2. Total nasal symptoms score 3. Sense of smell 4. Nasal polyp score by endoscopy 5. Peak nasal inspiratory flow rate 6. Serum eosinophil-derived neurotoxin 7. High-sensitivity C-reactive protein levels	Short oral steroid therapy followed by topical steroid therapy is significantly more effective over 6 months than topical steroid therapy alone in decreasing polyp size and improving olfaction in CRSwNP patients with at least moderate nasal polyps
Van Zele et al.	2010	1b	Prospective double-blind RCT	Adult CRSwNP patients treated with oral methylprednisolone (32 mg/day on days 1 to 5; 16 mg/day on days 6 to 10; 8 mg/day on days 11 to 20) (n = 14) or placebo (n = 19)	1. Polyps size measured by endoscopy 2. Nasal peak inspiratory flow 3. Blood analysis for eosinophils, eosinophilic cationic protein and soluble IL-5 receptor 4. Nasal secretion analysis for eosinophilic cationic protein, IL-5, IgE, matrix metalloproteinase-9, myeloperoxidase 5. Need for rescue surgery and need for rescue nasal steroids	Methylprednisolone significantly decreased nasal polyp size compared with placebo. The effect was maximal at week 3 and lasted until week 8. Methylprednisolone significantly reduced levels of ECP, IL-5, and IgE in nasal secretions

*RCT* randomized controlled trial, *CRS* chronic rhinosinusitis, *CRSwNP* chronic rhinosinusitis with nasal polyps

- Evidence level: A.
- Benefits–harm assessment: AE’s of systemic GCS outweigh advantages of therapeutic value in the long-term, except in patients with severe symptomatology.
- Recommendation: Strong recommendation against. Option for a short-term course in patients with severe symptoms and therapy-resistance.

A separate indication, for which oral GCS have been prescribed in CRSwNP patients, is the preoperative setting, in order to reduce perioperative bleeding and improve surgical conditions for the surgeon during endoscopic sinus surgery (ESS). Of the five studies that have been performed studying this topic in adults (Table 6), four are RCTs, however, their outcomes are not conclusive The study from Ecevit demonstrated a significant improvement on all perioperative variables studied (perioperative bleeding, visibility of the operative field, operative time, hospital stay) after a preoperative course of GCS in CRSwNP patients [59]. However, while some other studies confirm a significant improvement of intraoperative bleeding time [67] or quality of the operating field [68] and surgical time [69], these differences were not found to be significant by their colleagues [67–70]. A recent meta-analysis reported on a significant reduction in operating time, perioperative blood loss and improved surgical field quality when patients were given preoperative steroid treatment, however, the result was mainly based on a large RCT reporting on intranasal GCS [71]. Therefore, the use of oral GCS is currently not recommended in the preoperative setting of CRSwNP patients.

**Table 6:** Summary of the evidence for ‘efficacy of systemic steroids before endoscopic sinus surgery in CRSwNP’

Study	Year	LOE (1a to 5)	Study design	Study groups	Clinical endpoints efficacy	Conclusion
Ecevit et al.	2015	1b	Prospective double-blind RCT	Adults with CRSwNP with surgical indication receiving either oral prednisolone, 60 mg/day for 7 day, then reduced to 10 mg (1 tablet) taken every other day, stopping on d 17 (n = 11) or placebo (n = 10)	1. Perioperative bleeding 2. Visibility of the operative field 3. Operative time 4. Hospital stay	Perioperative bleeding, operative time and hospital stay were significantly reduced in patients who received oral steroids. Visibility of the operative field was significantly better after receiving steroids
Wright et al.	2007	1b	Prospective double-blind RCT	26 adult CRSwNP patients with surgical indication receiving either 30 mg of prednisone for 5 days preoperatively or placebo	1. Difficulty of surgery 2. Operative time 3. Peroperative blood loss	Surgeons rated the surgery in the placebo-treated group as more difficult than the steroid-treated group No differences were noted in operative duration and blood loss
Günel et al.	2015	1b	Prospective double-blind RCT	65 adult CRSwNP patients with surgical indication receiving either oral prednisolone (1 mg/kg for 2 days and then tapered down, with treatment completed on the day 10) or placebo	1. Intraoperative blood loss 2. Quality of surgical field	No difference in intraoperative blood loos when patients received oral steroids preoperatively Non-significant improvement of quality of surgical field after oral steroids
Fraire et al.	2013	3b	Prospective non-randomized CT	Adult CRS patients with surgical indication (CRSsNP and CSRwNP) receiving either 2×/day 30 mg methylprednisolone on 5 consecutive days prior to surgery (n = 27) vs. no treatment (n = 27)	1. Intraoperative bleeding 2. Surgical time 3. Quality of surgical field	Operative bleeding was significantly reduced in CRSwNP patients who received oral steroids preoperatively. No significance obtained in quality of operating field. No difference in surgical time
Sieskiewicz	2006	1b	RCT	36 adult CRSwNP patients with surgical indication receiving either prednisone (30 mg/day for 5 consecutive days directly before the surgery) or no preoperative treatment	1. Intraoperative bleeding 2. Surgical time 3. Quality of surgical field	Quality of the operating field and surgical time were significantly improved in CRSwNP patients who received oral steroids preoperatively. No significance obtained in total blood loss

*RCT* randomized controlled trial, *CRS* chronic rhinosinusitis, *CRSwNP* chronic rhinosinusitis with nasal polyps

- Evidence level: B.
- Benefits–harm assessment: AE’s of systemic GCS outweigh advantages of therapeutic value.
- Recommendation: Strong recommendation against.

### 6. Allergic fungal rhinosinusitis

Allergic fungal rhinosinusitis (AFRS) is a form of a non-invasive fungal rhinosinusitis and although it is not characterized by a specific phenotype, it seems to be an immunologically distinct subtype of CRS [72]. The diagnosis is based on the criteria proposed by Bent and Kuhn: (1) production of eosinophilic mucin without fungal invasion into sinonasal tissue; (2) positive fungal stain of sinus contents; (3) nasal polyposis; (4) characteristic radiographic findings; and (5) allergy to fungi [73]. In view of the locally aggressive character of the disease, the cornerstone of AFRS treatment is surgery [74]. However, a lot of uncertainty remains concerning the medical options and postoperative therapy. Although no RCTs are available, we found four smaller studies that investigated the role of GCS in the management of AFRS mostly in adults (Table 7). Two prospective non-controlled studies examined the effects of GCS in a small number of AFRS patients without surgery [75, 76]. Woodworth showed a significant reduction in nasal endoscopy scores and inflammatory markers in the AFRS group after 18 days of prednisone [76]. Landsberg [75] showed a more significant reduction in radiologic and mucosal scoring in AFRS patients compared to CRSwNP patients after 10 days of prednisolone. An older retrospective study from Kupferberg [77] in 26 AFRS patients, found that patients who received postoperative GCS showed more symptom improvement and less endoscopic disease compared to treatment with oral antifungals or no treatment. However, disease recurrence was noted after cessation of GCS. Similar findings were seen in a non-controlled retrospective study from Kuhn and Javer [78] who showed a maintenance of low endoscopic scores in AFRS patients, only after long-term GCS use. No AE’s were reported in any of the four studies. It has to be noted that all of these studies have a high risk of bias and the level of evidence for the use of oral GCS in AFRS patients remains at level C.

**Table 7:** Summary of the evidence for ‘efficacy of systemic steroids in allergic fungal rhinosinusitis’

Study	Year	LOE (1a to 5)	Study design	Study groups	Clinical end-point efficacy	Conclusion
Woodworth et al.	2004	3b	Prospective case control study	Adults with CRSwNP from which 8 AFRS en 6 eosinophilic mucin rhinosinusitis were treated with oral prednisone (60 mg for 3 days, 40 mg for 3 days, 30 mg for 3 days, 20 mg for 12 days)	1. SNOT-20 2. Nasal endoscopy score 3. Mucosal IL-5, IL-13, eotaxin, MCP-4	Significant reduction in nasal endoscopy scores and inflammatory markers, non-significant reduction in SNOT-20 scores
Landsberg et al.	2007	3b	Prospective case control study	Adult AFRS and CRSwNP patients received 1 mg/kg prednisone for 10 days	1. CT Lund Mackay scores 2. Nasal endoscopy score, but no scoring system used	CT score changes were significantly greater in AFRS patients compared to CRSwNP
Kupferberg et al.	1997	4	Retrospective case control study	Adult and adolescent AFRS patients (13–69 years) that underwent surgery and receiving: (1) no treatment; (2) oral steroids (4 days 40 mg, then 4 days 30 mg, then 20 mg/day until 1 month postop); (3) oral steroids and oral antifungals; (4) oral antifungals	1. Nasal endoscopy score 2. Symptom scoring	Postoperative treatment with oral steroids alone improved 90% of the patients, however, disease recurrence was seen after cessation of steroids
Kuhn and Javer	2000	4	Case series	Postoperative steroids in adult AFRS patients (0.4 mg/kg/day for 4 days, then 0.3 mg/kg/day for 4 days, then 0.2 mg/kg/day maintenance dose)	Nasal endoscopy score	Endoscopic stage 0 maintained if oral steroid was maintained for an average of 4.5 months

*CRS* chronic rhinosinusitis, *CRSwNP* chronic rhinosinusitis with nasal polyps, *AFRS* allergic fungal rhinosinusitis, *IL* interleukin, *MCP* monocyte chemotactic protein

- Evidence level: C.
- Benefits–harm assessment: Balance of harm and benefit in patients with severe disease.
- Recommendation: Option in patients with severe AFRS (severe symptoms and/or locally invasive disease) in conjunction with ESS.

### 7. Nasal manifestations of auto-immune disease

Many auto-immune disorders can involve the nose: thyroid auto-immunity, various vasculitis, Sjogren’s syndrome and sarcoidosis are the most frequently encountered, but other connective tissue diseases, such as systemic lupus erythematosus, polyarteritis nodosa, scleroderma and relapsing polychondritis can also have nasal symptoms [39].

GCS have been the major therapeutic option for some of these diseases as an immune suppressant for the past decades, probably being most effective where eosinophils, which are exquisitely steroid-sensitive, are involved [79]. However, the quality of the evidence for their efficacy is poor, with studies mostly being reviews or open pilots, even in seminal trials such as those of Fauci for Wegener’s granulomatosis [80–82]. The reasons for this include not only time-hallowed use, but also difficulty in undertaking placebo-controlled trials in severe diseases, differences in the manifestations and their intensity between individual patients, disease complexity and plasticity and probably lack of interest in funding. This situation is now changing with the advent of newer therapies, particularly monoclonal antibodies, which are being trialled against older therapies including GCS [83].

Churg–Strauss syndrome, now called eosinophilic granulomatosis with polyangiitis (EGPA), is classically considered a Th2-mediated disease and affects sino-nasal mucosa in > 80% of the patients. Treatment must be tailored according to prognostic factors identified by the French Vasculitis Study Group [84]. GCS alone are used for mild disease, high-dose GCS and cyclophosphamide is still the gold standard for severe cases [85], but biological agents such as rituximab or anti-IL-5 biologicals are promising, though costly, alternatives [86].

The hallmark of granulomatosis with polyangiitis (GPA; previously known as Wegener’s disease) is the coexistence of vasculitis and granuloma and again over 80% of patients show sino-nasal involvement [87]. GCS alone are insufficiently effective: the induction treatment for severe GPA comprises GCS combined with another immunosuppressant, cyclophosphamide or rituximab. Once remission is achieved, maintenance strategy following cyclophosphamide-based induction relies on less toxic agents such as azathioprine or methotrexate.

GCS decrease the frequency, duration, and severity of flares in relapsing polychondritis, but do not stop disease progression in severe cases [88].

The presence of sino-nasal disease is associated with more severe sarcoidosis and the need for systemic GCS therapy [89].

Treatment for systemic lupus erythematosus (SLE) by various organ systems is not evidence-based beyond the usual first- or second-line treatment, however a recent meeting achieved consensus in several scenarios, including anti-phospholipid syndrome [90].

GCS, often combined with NSAIDs, are used in Sjogren’s syndrome to treat associated interstitial lung disease and/or sensorineural hearing loss [91].

Table 8 shows the evidence available for auto-immune disorders for which GCS are frequently used.

**Table 8:** summary of the evidence for efficacy of systemic steroids in the treatment of auto-immune disease

Auto-immune disease + study	Year	LOE	Study design	Study groups	Conclusion
EGPA Moosig et al.	2013	3	A retrospective cohort study at a vasculitis referral centre	150 fulfilled the inclusion criteria. Of those, 104 had more than one follow-up visit severe organ manifestations: heart (46%), kidney (18%) and lungs (10%). Cyclophosphamide was used in 107 patients (71%). The prednisolone-doses of all patients were within the targeted range (i.e. ≤ 7.5 mg) in 69% of the total follow-up time; the median dose at end of follow-up was 5 mg/day	10-year survival rate was 89%, mortality comparable to the general population (SMR 1.29). Patients with cardiac failure had increased mortality (SMR 3.06)
GPA WGET Research Group	2005	1b	180-patient multicentre, placebo-controlled RCT examining the efficacy of etanercept in WGCT	Severe disease received cyclophosphamide and corticosteroids; limited disease received methotrexate and corticosteroids etanercept (25 mg twice weekly) or placebo was added to conventional therapy	Addition of etanercept did not lead to more sustained remissions; lower levels of disease activity; reduction in time to remission nor the number or relative risk of flares; nor fewer severe or life-threatening adverse events or deaths
Relapsing polychondritis McAdam et al.	1976	3	Review	159 reported cases, 23 those of the authors	Three-fourths of our patients required chronic corticosteroid therapy with an average dose of 25 mg per day of prednisone. Corticosteroids decrease the frequency, duration, and severity of flares, but do not stop disease progression in severe cases. Mortality rate 30 percent in our series and 22 percent in the other 136 reported cases
EGPA Ribi et al.	2008	2	RCT	72 patients with newly diagnosed EGPA (FFS of 0) treated with CS alone. At treatment failure or relapse, patients were randomized to receive 6 months of oral AZA or 6 pulses of CYC	93% achieved remission with CS alone, 35% relapsed, mainly during the first year of treatment. Among the 19 patients randomized to additional immunosuppression, 5 of 10 receiving AZA and 7 of 9 receiving pulse CYC achieved remission, P = NS Survival rates in all patients at 1 and 5 years were 100% and 97%, respectively. At the end of followup, 79% of the patients whose disease was in remission required low-dose CS therapy, mainly to control respiratory disease. CS-related adverse events were observed in 31% of the 72 patients
GPA Hoffman et al.	1992	3	An open-label pilot study of weekly low-dose methotrexate (MTX) plus glucocorticoids (GC) for treatment of patients with WG	Weekly administration of MTX (at a mean stable dosage of 20 mg) and GC in 29 WG patients	Marked improvement in 76%. Remission achieved in 69%. 7% improved but had intermittent smoldering disease that precluded total withdrawal of GC, and 17% had progressive disease within 2–6 months of starting the study treatment. Two patients who initially achieved remission later relapsed after GC discontinued. Of those who remain in remission (mean followup time 14.5 months), 72% have not required GC for a mean period of 10 months
Sarcoidosis Aubart et al.	2006	3	Retrospective single-center study	Twenty patients with histologically proven SNS (men/women, 7/13; mean age, 32 ± 9 year) were compared with control patients with sarcoid but without sinonasal (SN) involvement. Each patient was matched with 2 controls for the date of admittance in our institution	SNS had significantly more frequent and severe involvement of vital organs than controls, had a longer history of sarcoidosis, and required systemic treatment more frequently (100% vs. 57.7%, P < 0.001) and for a longer time (78 ± 42 months vs. 29 ± 18 months, P < 0.0001). Corticosteroids maintenance dosage was high (10.5 ± 6 mg daily) and mainly depended on SN involvement
GPA Guillevin et al.	1997	2	Prospective multi-centre RCT	50 newly diagnosed WG patients every patient received a daily injection of methylprednisolone for 3 days, followed by daily oral prednisone (1 mg/kg/day) and a 0.7-g/m^2^ pulse of CYC. Patients were then randomly assigned to prednisone plus intravenous pulse CYC (group A), n = 27 or prednisone plus oral CYC (group B) n = 23 as first-line treatment	Pulse CYC was as effective as oral CYC in achieving initial remission of WG with fewer side effects and lower mortality. However, in the long term, treatment with pulse CYC does not maintain remission or prevent relapses as well as oral CYC

*EGPA* eosinophilic granulomatosis with polyangiitis, *GPA* granulomatosis with polyangiitis, *AZA* azithromycin, *CYC* cyclophosphamide

- Evidence level: D.
- Benefits–harm assessment: Depending on other organ involvement and severity.
- Recommendation: Following the recommendation for the management of the specific auto-immune disease.

### 8. Sino-nasal pathology and concomitant asthma

Asthma is a chronic inflammatory disease of the lower airways involving inflammation of the bronchial mucosa, and variable obstruction of bronchi due to intrinsic/extrinsic stimuli, and leading to symptoms such as episodic breathlessness and wheezing with airway hyperresponsiveness to environmental stimuli [92]. Since the introduction of the “United Airway Disease” concept [1], a large series of scientific publications from clinical epidemiology, pathophysiology, histology, and treatment outcomes has correlated asthma and upper airway disease. AR and asthma often coexist and AR is regarded as a risk factor for the development of asthma. Uncontrolled rhinitis impacts asthma control. Asthmatic patients have a higher CRS severity score than non-asthmatic patients, and more nasal polyps, indicative of a strong relationship between CRS severity and asthma [93]. It has been reported that 20–60% of patients with CRSwNP have asthma [94, 95].

The first use of GCS to treat acute asthma exacerbation was in 1956 [96]. Development of GCS that have less mineralocorticoid activity, like prednisone, and later those that have no mineralocorticoid activity, like dexamethasone, made steroid use more attractive therapies to use in asthma. Prescribing a short course of oral GCS following the treatment of acute asthma exacerbations was found to reduce the rate of relapse [97]. However, courses longer than 5 days were not found to provide any additional benefit [98].

As described above, systemic GCS should not be considered as a treatment for AR. We could not identify any systematic review, randomized trial, or controlled study that evaluated the use of systemic GCS in patients with AR with concomitant asthma not responding to other therapy.

When analysing the evidence of oral GCS for patients with CRS and coexisting asthma there are a few randomized controlled trials and uncontrolled prospective interventional studies that evaluated the efficacy of different treatments (Table 9) of which only one looked at systemic GCS use. This study was carried out in adults by Ikeda et al. [99] and included 21 CRS patients with concomitant asthma. Fifteen patients underwent ESS, and 6 other patients remained on medical therapy. Seven patients of the ESS group showed a reduction in the need for GCS during the 6 months following surgery, whereas two patients were unchanged and two patients required larger dosages.

**Table 9:** Summary of the evidence for ‘efficacy of systemic steroids in sinonasal disease + concomitant asthma

Study	LOE (1a to 5)	Study design	Study groups	Clinical endpoints efficacy	Conclusion
Ikeda et al. [99]	3	Prospective RCT	Adult CRSwNP and CRSsNP patients undergoing ESS	1. Sinonasal and pulmonary symptoms 2. Systemic GCS need	1. Improvement of FEV_1_ 2. No significant changes in systemic GCS need

*RCT* randomized controlled trial, *CRS* chronic rhinosinusitis, *CRSsNP* chronic rhinosinusitis without nasal polyps, *CRS* chronic rhinosinusitis, *CRSwNP* chronic rhinosinusitis with nasal polyps, *GCS* glucocorticosteroids

Generally, due to a lack of studies investigating the efficacy of GCS in asthmatics with CRS, the same rules apply as for non-asthmatic CRS patients. With regards to the morbidity and potential mortality that is associated with asthma, the use of GCS in asthmatic CRS patients should be directed in the first place by the severity of the lower airway symptoms.

- Evidence level: D.
- Benefits–harm assessment: AE’s of systemic GCS outweigh advantages of therapeutic value in the long-term, except in patients with severe symptomatology.
- Recommendation: Recommendation against. Option in patients with severe symptoms and therapy-resistance.

## Adverse effects of systemic GCS

Although GCS play a key role in the treatment of various inflammatory disorders, including chronic upper airway disease, a quite extensive range of potential AE’s is well-described in literature and the chance to develop these effects seems to increase with higher dose and longer duration of treatment [8, 100–102].

However, few studies have actually addressed the risk of common GCS-induced AE in upper airway disease. Also, most of the studies available on GCS focus on high dose or long-term usage for at least 6 months or even 1 year consecutively, which is mostly less relevant in the upper airway disease patient group.

In the following section, we aimed at summarizing the data of potential short- as well as long-term AE’s of systemic GCS treatments for rhinitis and/or rhinosinusitis in the adult population. Due to the heterogeneity in studies, treatment regimens and patient populations, we classified the side-effects according to the organ-system involved, but no further subdivision was made. When no studies were available for upper airway disease patients, a mention of studies investigating AE’s in similar patients (ophthalmologic, asthmatic) was made. Studies investigating side-effects in children will be discussed separately in the next chapter.

### 1. Hypothalamic–pituitary–adrenal-axis (HPA) inhibition

Reductions in the level of plasma cortisol are reported after one injection of GCS. They usually decrease in the first 2 weeks after steroid administration, but slowly return to normal after 3 weeks, as has been demonstrated in patients with AR [103]. Hedner et al. [104] showed a minor HPA dysfunction in 14 allergic patients treated with a single intra-muscular injection of MP acetate, which returned completely to normal at 4 weeks post-injection. In a double-blind study by Laursen et al. [105] 36 birch pollen allergic patients were treated with either a single injection of betamethasone dipropionate or oral prednisolone 7.5 mg/day for 3 weeks. Only the prednisolone treated patients showed reduction in plasma cortisol levels at 3 weeks.

Bonfils et al. [106] prospectively evaluated the HPA-axis in patients with CRSwNP (n = 46), who received at least three short courses of oral GCS in the last year (course 6–8 days, 1 mg/kg/day, mean duration of treatment 4.7 years, mean 6.8 courses/year, mean cumulative prednisone consumption 3,800 mg). The study demonstrated that 48% of patients had an asymptomatic adrenal insufficiency diagnosed with the Synacthen test.

### 2. Hyperglycemia and diabetes

A retrospective study based on Danish National Registries, including 47,382 AR patients, demonstrated that treatment with at least one consecutive injection of depot corticosteroid for 3 years on a row was associated with an increased risk of being diagnosed with diabetes later in life (RR 1.4) [107]. The degree of new-onset diabetes associated with intermittent short-term oral GCS has not been clearly established.

### 3. Osteoporosis

In the same Danish epidemiological study, Aasbjerg et al. [107] showed that, compared to immunotherapy, treating AR with annual depot-steroid injections (i.e. at least one steroid injection in the pollen season for 3 consecutive years) was associated with increased risk of being diagnosed with osteoporosis (RR 1.2). The above-mentioned study from Bonfils, investigating the HPA-axis, prospectively evaluated the occurrence of osteoporosis in patients with CRSwNP (n = 46), receiving at least three short courses of oral GCS in the previous year. Osteopenia of the proximal femur was present in 40.5% and osteoporosis was present in 54% [106]. Rajeskaran et al. [108] retrospectively evaluated the risk of osteoporosis in patients with CRS (n = 176), who received oral GCS ≥ 5 mg daily for 3 consecutive months any time in the past. Overall, low bone mineral densities (BMD; osteopenia or osteoporosis) was 38.6%. These studies were recently evaluated in a systematic review which was unfortunately not able to quantify the overall risk of osteoporosis induced by oral GCS for CRSwNP, due to the low number of studies [109].

The effects of short-course oral GCS on bone mineral density (BMD) have also been investigated in a 4-year longitudinal small study in asthmatic patients. Asthmatic patients receiving frequent short courses of oral GCS (i.e. > 2.5 courses/year; n = 9) compared to those receiving sporadic courses (i.e. ≤ 2.5 courses/year; n = 26) revealed a greater loss of lumbar BMD (T-score 82.0% versus T-score 77.7%) in the frequently treated group [110]. Also, a lower Z-score of 93.1% was demonstrated in frequent short courses, versus the sporadic courses that did not show a lower Z-score than the normal population values (Z-score 100.1%).

### 4. Avascular necrosis

With regards to avascular necrosis of the femoral head in patients treated with systemic GCS for upper airway disease, we found 1 case report of Nasser et al. [111] describing a single case with severe hay fever that was given at least one depot corticosteroid injection each year for 11 years, leading to avascular necrosis.

More individual case reports highlight the relationship between the use of systemic GCS and avascular necrosis. The risk to develop osteonecrosis seems to be dependent on the prescribed dose, the cumulative dose and route of administration, as well as underlying disease states (SLE patients seem to be particularly at risk) [112–114].

### 5. Gastrointestinal disturbances and peptic ulceration

In a randomized double-blind placebo-controlled study by Kirtsreesakul et al. [62] 112 patients with CRSwNP used either 50 mg prednisone or placebo for 14 days and reported significantly more (mild) gastrointestinal disturbances and dyspepsia in the prednisolone treated group. In a double-blind placebo-controlled trial by Venekamp et al. [41] 174 adult patients clinically diagnosed with ARS received either 30 mg/day prednisolone or placebo for 7 days. The incidence of gastrointestinal complaints did not differ between treatment groups.

In a large nested case–control analysis based on the UK General Practice Research Database, 2105 cases of upper gastro-intestinal complications were compared to 11,500 controls and then evaluated for exposure to certain drugs e.g. corticosteroid use. The adjusted OR for current use of oral GCS was 1.8 (95% CI 1.3–2.4) for upper gastrointestinal complications overall [115]. No statistically significant difference could be objectified for lower versus higher dosage of GCS. To our knowledge no studies in upper airway disease patients report on systemic steroid treatment and peptic ulceration.

### 6. Ocular adverse effects

GCS have been described to induce the formation of posterior subcapsular cataract or glaucoma. The risk for patients using repeated (short) courses of systemic GCS for upper airway disease is currently unknown.

There is evidence in rheumatoid arthritis patients that this risk is enhanced after therapy lasting more than 1 year [116]. Another study by Huscher et al. [101] analysed dose-related patterns of self-reported symptoms from 1066 patients with RA with ongoing long-term (> 6 months) systemic GCS. These symptom patterns were compared to non-users (no systemic GCS for at least 12 months). The prevalence of self-reported cataract was higher for all dosages of GCS, whereas the prevalence of self-reported glaucoma was only increased in those taking > 7.5 mg/day (6.6% users vs. 2.7% non-users).

### 7. Infections

A meta-analysis of randomised controlled clinical trials in which patients were randomised to treatment with or without systemic GCS (n = 4198) showed that the rate of infection was not significantly increased in patients who were given a mean dose of less than 10 mg/day of prednisone or a cumulative dose of less than 700 mg [117]. This meta-analysis included a wide variety of diseases warranting systemic GCS. The true risk of developing infection in patients using short courses for upper airway disease remains uncertain.

### 8. Local adverse effects of steroid-injections

We found one case report on gluteal subcutaneous atrophy that was seen after a depot steroid injection of triamcinolone for AR [118]. A study of Laursen et al. [34] investigated specifically the reporting of all AE’s related to GCS injections for AR to the ‘Danish Register for the Side-Effects of Drugs’ and evaluated the reported events consecutively for a 10-year period. The study demonstrated that one out of 11,785 injections came with any local AE. Most AE’s were reversible and primarily skin related, such as skin atrophy.

### 9. Cardiovascular adverse effects

Cardiovascular disease is mainly associated with high dose and long-term use, primarily hypertension and acute myocardial infarction are described [100, 119].

A population-based cohort study in 68,781 GCS users and 82,202 non-users showed that patients exposed to dosages of GCS > 7.5 mg of prednisolone/day (or equivalent) during 1 to 5 years of follow-up, had substantially higher rates of myocardial infarction, heart failure, or cerebrovascular disease (adjusted RR of 2.56; 95% CI 2.18–2.99). The risk was not increased in patients using < 7.5 mg prednisolone equivalent daily [120].

Another large, retrospective case–control study with data extracted from the General Practice Research Database (1988–1997) showed in over 100,000 individuals that the use of oral GCS comes with a 25% higher risk of any cardiovascular or cerebrovascular outcome compared to controls. Current use (in the 3 months before the registration of an event) and highest average daily dose give a much stronger association. Current use is also associated with a significantly increased risk of heart failure (adjusted OR of 2.66; 95% CI 2.46–2.87) and ischemic heart disease (OR of 1.20; 95% CI 1.11–1.29), but not ischemic stroke or transient ischemic attack. Cardiovascular risk showed a clear dose–response relationship [121].

To our knowledge, the risk in patients using GCS for intermittent short courses is unknown.

### 10. Neuropsychiatric effects

A study from Hissaria et al. [60] investigating 40 CRSwNP patients treated with 50 mg of prednisolone daily for 14 days or placebo, found that sleep disturbances were reported as a significant prevalent AE (40%) compared to placebo (10%). Mood disturbance were more frequently reported, but not significantly different from placebo (25% vs. 10%).

In the above-mentioned controlled trial by Venekamp et al. [41] studying ARS patients treated with 30 mg/day prednisolone or placebo for 7 days, the incidence of mood or sleep disturbance did not differ between treatment groups.

Two studies in asthmatic and ophthalmologic patients receiving short-courses of GCS, showed a development of (hypo)mania [122, 123] as well as depression symptoms [123].

Naber et al. [123] showed in a prospective uncontrolled study in ophthalmologic patients receiving systemic GCS (n = 50) that 26–34% of patients developed (hypo)mania and 10–12% developed depression syndromes when using an initial 119 ± 41 mg/day MP or fluorcortolone, tapered to 75 ± 22 mg/day at 8 days. The onset of symptoms was within 3 days of use and there was no correlation between daily dose and daily ratings of mood. Brown et al. [122] showed in 32 asthmatic patients using prednisone (mean course 13.9 days, mean dose of 36.9 mg/day) a highly significant increase in self-reported mania, but no increase in depression during the first 3–7 days of therapy. Mood changes returned back to normal after discontinuation of therapy.

### 11. Cushingoid features

We found no studies investigating Cushingoid appearance in rhinitis/rhinosinusitis patients treated with GCS and only a few studies addressed the risk of intermittent short courses of GCS and weight gain.

A randomised controlled trial by Campieri et al. [124] in patients with active Crohn’s disease demonstrated that 38% of patients on a regimen of prednisolone tapered over 12 weeks (40–45 mg) developed a ‘moon face’. Mean body weight increased with 2.1 kg after 8 weeks of treatment. Bar-Meir et al. [125] showed that patients receiving 8 weeks of prednisone developed a moon face in 33% versus 16% in patients receiving a similar treatment with budesonide.

## Benefit and risk of use of GCS in pediatric populations

Inflammatory diseases of the nose and paranasal sinuses in children include upper respiratory tract infections, chronic rhinitis, ARS and CRS. ARS is defined as increase of sinonasal symptoms after 5 days of infection or persistent symptoms after 10 days and characterized by the sudden onset of two or more of the symptoms (discoloured nasal discharge, nasal blockage/obstruction/congestion, cough at daytime and night-time) for less than 12 weeks [4]. Bacterial infection is expected when at least 3 symptoms are present among which discoloured discharge, purulent secretion in nasal cavity, severe local pain with a unilateral predominance, fever, elevated C-reactive protein or erythrocyte sedimentation rate, and double sickening (i.e. deterioration after an initial milder phase of illness) [4]. The definition of pediatric CRS differs from adult CRS by the symptom of cough [4] and is defined by the presence of two or more symptoms, one of which should be either nasal obstruction or nasal discharge (anterior or posterior) with/without facial pain/pressure with/without cough, lasting for at least 12 weeks [4]. The diagnosis is confirmed by either nasal endoscopy showing edema, purulent drainage or nasal polyps in the middle meatus or CT scan showing ostiomeatal complex or sinus opacification. Of note, the presence of nasal polyps is much less common in pediatric patients than in adult patients with CRS [126].

### 1. Efficacy of systemic GCS in pediatric CRS and ARS

Three clinical trials can be found in literature that investigated the use of oral GCS in the pediatric rhinosinusitis population, of which only one is controlled (Table 10).

**Table 10:** Summary of the evidence for ‘efficacy of systemic steroids in pediatric sinonasal disease’

Study	Year	LOE (1a to 5)	Study design	Study groups	Clinical end-point efficacy	Conclusion
Ozturk et al.	2011	1b	RCT	1. Children with CRSsNP (6–17 years) receiving antibiotics and methylprednisolone 1 mg/kg and reduced progressively over a 15-day treatment course 2. Children with CRSsNP receiving antibiotics and placebo	1. Change in mean symptom and CT scores pre- and post-treatment 2. Change in individual symptom scores, relapse rate	Beneficial effect of MP in combination with antibiotics on mean symptoms, CT scores, VAS for cough, nasal obstruction and post-nasal drainage. No difference in relapse rate
Scorpinski et al.	2008	3b	Retro-spective uncontrolled	1741 children with CRS treated with antibiotics, intranasal topical corticosteroids and oral corticosteroids (> 4 days) or combination	CT scores	Improvement of CT scores after oral corticosteroid treatment, in mono- or pluritherapy
Tosca et al.	2003	4	Uncontrolled prospective cohort study	30 asthmatic CRS children treated with antibiotics, intranasal steroids and a short course of deflazacort (1 mg/kg daily for 2 days, 0.5 mg/kg daily for 4 days and 0.25 mg/kg daily for 4 days)	Nasal endoscopy and cytokine patterns in nasal lavages	Resolving of purulent discharge after combination treatment and decrease of mean IL4-levels in nasal lavage

*RCT* randomized controlled trial, *CR* chronic rhinosinusitis, *CRSsNP* chronic rhinosinusitis without nasal polyps, *CRS* chronic rhinosinusitis. *CRSwNP* chronic rhinosinusitis with nasal polyps, *MP* methylprednisolone

This controlled study involved 48 children (mean age 8 years) with CRSsNP [66] and investigated the effect of oral GCS as an add-on to antibiotics. 22 participants received either 30-day course of oral amoxicillin–clavulanate and 15-day course of oral MP and 23 participants received only antibiotics and a placebo. The mean change of total symptom score and CT score was significantly higher after treatment with oral GCS and antibiotics compared with placebo and antibiotics (P < 0.001). There was also a significant beneficial effect of oral GCS in cough, nasal obstruction and post-nasal drainage symptom scores. Complete clinical recovery after 30 days of treatment was obtained in significantly more subjects receiving MP (P < 0.005). Recurrence of symptoms 6 months after the end of treatment was not statistically significant between the groups.

Additionally, a retrospective study involving 35 young CRS patients (1–21 years) undergoing serial sinus CT scans due to medical reasons, evaluated Lund Mackay ostiomeatal complex score in relation to three different treatment schemes [127] antibiotics, intranasal topical GCS and oral systemic GCS. The data suggested that the use of systemic GCS was associated with a significant increase in the likelihood of radiologic improvement. The retrospective study design, the small and heterogeneous population, heterogeneous treatment modalities, and the lack of adjustments, limit the possibilities to assess clinical significance of the findings.

A second uncontrolled study [5] evaluated cytokine pattern of 30 asthmatic CRS patients (4–12 years) before and after the treatment of amoxicillin–clavulanate, fluticasone propionate aqueous nasal spray and a short course of oral deflazacort. After the treatment, endoscopic resolving of mucopurulent discharge was detected in 25/30 children, the median concentration of IL-4 decreased significantly in all subjects, and the median IFN-γ concentration increased significantly only in the atopic subgroup (N = 16). The uncontrolled study design and uncertainty whether the patients used prescribed drugs, limits the possibilities to assess effect of systemic GCS.

### 2. Harm of GCS in children

There is limited knowledge of risks of using systemic GCS in pediatric CRS or ARS compared to pediatric asthma. As an example, the Childhood Asthma Management Program trial followed the annual bone mineral accretion of 877 children (5–12 years) with mild-to-moderate asthma [128, 129]. Oral GCS bursts produced a dosage-dependent reduction in bone mineral accretion (0.052, 0.049, and 0.046 g/cm^2^ per year) and an increase in risk for osteopenia (10%, 14%, and 21%) for 0, 1–4, and ≥ 5 courses, respectively, in boys. The authors conclude that multiple oral GCS bursts over a period of years can produce a dosage-dependent reduction in bone mineral accretion and increased risk for osteopenia in children with asthma. 780 children with asthma were followed for a mean of 4.3 years and it was shown that boys with lower vitamin D levels are significantly more susceptible to the negative effects of GCS on bone mineral accretion over time [129]. Regarding studies investigating GCS AE’s in upper airway disease, the trial from Ozturk also looked at self-reported AE’s during the 15-day course of oral MP [66]. In this trial no clinically significant AE’s were reported. At the end of the treatment, the mean weight change did not differ statistically significantly between the groups. No data of monitored AE’s, nor that of long-term outcomes, nor that of bacterial culture were available in this study.

A systematic review has been performed to determine the most common and serious drug-related AE of long courses of oral GCS in children [130]. Literature search of several databases was performed to identify all studies in which systemic GCS had been administered to pediatric patients ranging from 28 days to 18 years of age for at least 15 days of treatment. The group found 91 studies that represented a total of 6653 children and contained reports of 4124 adverse drug reactions, the majority in patients with leukaemia, haemangioma and asthma. The three most frequent adverse drug reactions were weight gain (22.4%), Cushingoid features (20.6%) and growth retardation (18.9%). Increased susceptibility to infection was the most serious adverse drug reaction. 24 children died from infections, 10 from varicella zoster.

There is insufficient knowledge of the effect and harm of short-term systemic GCS courses in pediatric CRS patients. However, based on studies on pediatric asthma, a single short-term systemic GCS course could be considered in pediatric patients suffering from CRS that is not responding to other therapies such as intranasal GCS, antibiotics, supporting therapy (saline douchings, decongestants) and adenoidectomy. It is mandatory to perform more powered; randomized placebo-controlled clinical trials of pediatric ARS and CRS with long-term follow up and report of AE’s.

- Evidence level: B.
- Benefits–harm assessment: AE’s of systemic GCS outweigh advantages of therapeutic value in mild and moderate disease.
- Recommendation: Strong recommendation against. Option in patients suffering from very severe and therapy-resistant disease, in combination with antibiotics.

## Health economic considerations related to GCS use

Besides clinical consequences, systemic GCS use may also have some health economic implications that should be considered in its benefit-harm trade-off. Generally, the direct costs for systemic GCS are among the lowest quartile of prices of medications available worldwide. However, the indirect costs due to adverse events of (especially long-term, high-dose) systemic GCS use could be more substantial. Two industry-funded studies have assessed the cumulative economic burden of GCS associated adverse events regardless of dose, duration or indication [131, 132]. Manson et al. [131] identified 63 studies in which 21 different GCS adverse events were reported with increased fracture risk, gastric and psychiatric conditions being the most frequent ones. Their economic analysis from the UK perspective revealed that taking oral GCS would result in an additional annual cost of at least £165 for treatment of all steroid related adverse events. One study specifically assessed the economic impact of oral GCS on related fractures where hip, vertebral and forearm fractures costed £10,761, £1976 and £863 respectively. Notably, only three studies focused on patients with allergic rhinitis and/or skin diseases and none specifically on rhinosinusitis. A second review [132] included 47 studies reporting on adverse events of systemic GCS. Subsequently, a cost analysis was undertaken from the US perspective. It was unclear whether any patients with allergic rhinitis or rhinosinusitis were included. Most frequently reported adverse events were psychiatric and gastric conditions, infections and fractures. The authors estimated the potential cost reductions if the daily GCS dose would be reduced. Regarding avoidance of fractures, they estimated that 96 fractures per 10,000 elderly patients could be avoided summing up to $1.76 million ($176 per patient). The findings from both reviews should be interpreted with caution given the heterogeneous and often low-quality and retrospective nature of the studies included and the difficulty in excluding confounding due to underlying disease activity. Besides these two reviews with no particular disease focus, some studies focused on the costs of systemic GCS related adverse events within a specific population such as asthma [133, 134] or rheumatologic diseases [135, 136] and found increased costs in the GCS exposed populations. None were specifically focusing on rhinitis or rhinosinusitis. We conclude that given the limited amount of current evidence, more studies on the economic burden and cost-effectiveness of systemic GCS use in rhinitis and rhinosinusitis treatment are required.

## Alternatives for GCS in upper airway disease

In both rhinitis and rhinosinusitis patients, systemic GCS treatment is in general reserved for those in whom disease control cannot be obtained by baseline medical therapy (intranasal steroids and antihistamine/antileukotrienes for AR [30] and intranasal steroids and antibiotics for ARS/CRS [4]). However, in AR, allergen immunotherapy (AIT) is an alternative option for patients suffering from uncontrolled symptoms. AIT modifies the natural disease course and recent well-performed trials have demonstrated reductions in both symptoms and use of rescue medication in patients with AR for both the subcutaneous as well as sublingual administration route [137]. One study from 1969 compared the efficacy of one depot MP injection with a pre-seasonal administration of an alum-precipitated pyridine extracted grass pollen immunotherapy and found similar results between the two groups in terms of symptom improvement [138]. However, this paper already stated that the potential AE’s of MP do not justify the use of systemic GCS for a condition such as AR. One large Danish registry study including almost 40,000 AR patients actually showed the oral steroid-sparing effect of subcutaneous AIT (SCIT) for seasonal AR with an annual mean of 1.0 steroid injections in patients receiving SCIT versus a mean of 1.6 injections in the non-SCIT group. Of the SCIT-treated individuals, 84% did not need GCS at all after SCIT treatment [139]. Aasbjerg looked at the same registry to compare AE’s and found that AR patient treated with systemic GCS showed more diabetes and osteoporosis than those treated with AIT as mentioned above [107].

For CRS patients, current alternatives for oral GCS during exacerbations consist of antibiotics and when patients remain uncontrolled, sinus surgery is the next step in line [4]. However, studies investigating biological agents that are available for the treatment of asthma and/or other allergic diseases, have shown very beneficial effects in CRSwNP patients [140] but are currently only available for those with severe concomitant asthma.

Gevaert et al. [141] extrapolated results from different studies to compare the efficacy of different treatments in CRSwNP patients. They found a beneficial effect on NP score of doxycycline that was comparable to MP after 8 weeks. Also, omalizumab and mepolizumab treatment had better results on NP score than the oral GCS treatment. Omalizumab and mepolizumab additionally showed better symptom control compared to MP. Currently only data on the oral steroid-sparing effects of mepolizumab and benralizumab in asthma are available [142], but with the increased implementation of these therapies in CRSwNP, studies evaluating the steroid-sparing effect for upper airway exacerbations will be necessary.

## Conclusion

When disease control in upper airway disease cannot be obtained with intranasal steroids or other medical treatment prescribed by the respective guidelines, severe cases of AR, ARS, AFRS and CRSwNP can be treated with a short-term course of systemic GCS to improve symptoms. This manuscript provided an overview of the current evidence for the beneficial effects of systemic GCS in the different subtypes of upper airway diseases, as well as in the pediatric age group and aimed at providing recommendations for the specific disease entities.

However, multiple AEs have been widely described and therefore physicians should be aware of the risks associated with oral GCS and make a good risk–benefit assessment prior to prescribing them. In this paper, we summarize these potential AEs; given the current evidence in literature, a clear assessment of the risks associated with oral steroid use in upper airway disease cannot be made. Currently available data show a wide variability in diseases, patients, duration of treatment and follow-up and therefore this topic needs to be addressed in a systematic way in order to provide a substantiated recommendation for the use and dosing of oral GCS in the upper airway disease population.

We can conclude that, although some beneficial effects of systemic GCS have been demonstrated in chronic upper airway diseases such as AR and CRSwNP, systemic GCS should not be considered as a first line of treatment for these disease types.

## Supplementary information

**Additional file 1.** Search terms.

## Data Availability

Not applicable.

## Abbreviations

AFRS: allergic fungal rhinosinusitis
AE: adverse effect
AIT: allergen immunotherapy
AR: allergic rhinitis
ARS: acute rhinosinusitis
BMD: bone mineral density
CRS: chronic rhinosinusitis
CRSwNP: chronic rhinosinusitis with nasal polyps
CRSsNP: chronic rhinosinusitis without nasal polyps
EGPA: eosinophilic granulomatosis with polyangiitis
ESS: endoscopic sinus surgery
GCS: glucocorticosteroids
GM-CSF: granulocyte-macrophage colony-stimulating factor
GPA: granulomatosis with polyangiitis
GRα/β: glucocorticoid receptor α/β
GRE: glucocorticoid-responsive elements
HPA: hypothalamic–pituitary–adrenal-axis
ICAM: intercellular adhesion molecule
IFN-γ: interferon γ
IL: interleukin
INS: intranasal steroids
MP: methylprednisolone
NAR: non-allergic rhinitis
NP: nasal polyps
NSAID: non-steroidal anti-inflammatory drug
OR: odd’s ratio
RCT: randomised controlled trial
RR: relative risk
SCIT: subcutaneous immunotherapy
SLE: systemic lupus erythematosus
TNF-α: tumor necrosis factor α
VCAM: vascular cell adhesion molecule
