# Correction to: Benefits and harm of systemic steroids for short- and long-term use in rhinitis and rhinosinusitis: an EAACI position paper

**DOI:** 10.1186/s13601-020-00343-w

**Published:** 2020-09-28

**Authors:** Valerie Hox, Evelijn Lourijsen, Arnout Jordens, Kristian Aasbjerg, Ioana Agache, Isam Alobid, Claus Bachert, Koen Boussery, Paloma Campo, Wytske Fokkens, Peter Hellings, Claire Hopkins, Ludger Klimek, Mika Mäkelä, Ralph Mösges, Joaquim Mullol, Laura Pujols, Carmen Rondon, Michael Rudenko, Sanna Toppila-Salmi, Glenis Scadding, Sophie Scheire, Peter-Valentin Tomazic, Thibaut Van Zele, Martin Wagenmann, Job F. M. van Boven, Philippe Gevaert

**Affiliations:** 1 Cliniques Universitaires Saint-Luc Brussels, Av. Hippocrate 10, 1200 Brussels, Belgium; 2 Department of Otorhinolaryngology, Amsterdam University Medical Centres, AMC, Amsterdam, The Netherlands; 3 Upper Airway Research Laboratory, Dep. of Otorhinolaryngology, Ghent University Hospital, Ghent, Belgium; 4 Bispebjerg University Hospital, Copenhagen, Denmark; 5 Faculty of Medicine, Transsylvania University, Brasov, Romania; 6 Hospital Clínic, IDIBAPS, CEBERES, Universitat de Barcelona, Catalonia, Spain; 7 Centro Medico Teknon, Barcelona, Spain; 8 Department of Ear, Nose and Throat Diseases, Karolinska University Hospital, Stockholm, Sweden; 9 Pharmaceutical Care Unit, Faculty of Pharmaceutical Sciences, Ghent University, Ghent, Belgium; 10 Allergy Unit, Hospital Regional Universitario of Málaga, IBIMA, ARADyAL, Malaga, Spain; 11 Department of Ear, Nose and Throat Disease, University Hospitals, Louvain, Belgium; 12 ENT Department, Guy’s & St Thomas’ Hospital, London, UK; 13 Center of Rhinology and Allergology, Wiesbaden, Germany; 14 Skin and Allergy Hospital, Helsinki University Hospital and University of Helsinki, Helsinki, Finland; 15 University of Cologne, Cologne, Germany; 16 London Allergy and Immunology Center, London, UK; 17 Royal National Throat, Nose and Ear Hospital, London, UK; 18 Medical University Graz, Graz, Austria; 19 Heinrich-Heine-University, Düsseldorf, Germany; 20 Department of Clinical Pharmacy & Pharmacology, University Medical Center Groningen, Groningen Research Institute for Asthma and COPD (GRIAC), University of Groningen, Groningen, The Netherlands

## Correction to: Clin Transl Allergy (2020) 10:1 10.1186/s13601-019-0303-6

Following publication of the original article [1], the authors identified an error in the author name of Martin Wagenmann.

The incorrect author name is: Martin Wagemann.

The correct author name is: Martin Wagenmann.

The author group has been updated above and the original article [1] has been corrected.
