# Supplementary material for: Benefits and harm of systemic steroids for short- and long-term use in rhinitis and rhinosinusitis: an EAACI position paper
Source: Clin Transl Allergy. 2020 Jan 3;10:1. doi: 10.1186/s13601-019-0303-6 (PMC6941282; doi:10.1186/s13601-019-0303-6)
Supplement: Supplementary file 1 — Additional file 1. Search terms. [file 13601_2019_303_MOESM1_ESM.docx]

Benefits and Harm of systemic steroids for short- and long-term use in rhinitis and rhinosinusitis – an EAACI position paper.

Additional file 1.

**Search terms**

Search terms on diseases included rhinitis or rhinoconjunctivitis or rhinosinusitis or sinusitis or “nasal polyps” or polyposis. Search terms on treatment included corticosteroid or corticoid or glucosteroid or glucocorticoid or corticotherapy or methylprednisolone or medrol or prednisone or prednisolone methasone or celestone or dexamethasone, coupled with oral or intramuscular or systemic or injection or depot or intravenous.

Search strategy for the pediatric population included all of the above and was coupled with pediatric or paediatric or children or young or adolescents.

Search terms on adverse events were the following:

- long term adverse effects or "drug-related side effects and adverse reactions" or "metabolic side effects of drugs and substances" or adverse drug reaction reporting systems or pharmacovigilance or safety or patient harm or patient safety

- iatrogenic disease

- pharmacovigilance or vigilance or FDA or harm alert

- (adverse or side effect or ((longterm or long-term) complicat) or sequel or harm or safe or safety or toxic or iatrogen

- (corticosteroid or corticoid or steroid or glucosteroid or glucocortico or methylpred or medrol or metipred or urbason or prednis or prednime or encortn or desoxycorticosteron or betamethason or celeston or budesonid or Pulmicort or Rhinocort or dexamethason) adj (induced or associated or related or harm)

- ((complicat or adverse of side effect or harm or toxic) adj (corticosteroid or corticoid or steroid or glucosteroid or glucocortico or methylpred or medrol or metipred or urbason or prednis or prednime or encortn or desoxycorticosteron or betamethason or celeston or budesonid or Pulmicort or Rhinocort or dexamethason)) and (long-term or longterm or chronic or prolonged or continous or oral or inhal or depot or intramuscul)

- ((risk or harm) adj3 benefit) and (long-term or longterm or prolonged or (long adj period) or ((long or continuous or chronic or frequent or months or weeks or regimen) adj3 ("use" or usage)) or oral or systemic or inhal or intravenous or intramuscul or depot or iv or ICS)

*Coupled with*

*-* (adrenal cortex hormones or exp glucocorticoids) and (adrenal cortex hormones to or exp glucocorticoids)

- (methylpred or medrol or metipred or urbason or prednis or prednime or encortn or desoxycorticosteron or betamethason* or celeston* or budesonid* or Pulmicort or Rhinocort or dexamethason* or (systemic adj3 triamcinolon*) or Kenacort

|  |
| --- |
| ( |
| ((corticosteroid* or corticoid* or steroid or steroids or glucosteroid* or glucocortico* or corticotherap* or cortico-therap*) not (non-steroid* or (((corticost* or steroid*) adj inject*) or ((sex or gonadal or hormon*) adj2 steroid*) or ((steroid or corticoster* or glucocorticoid*) adj (responsive* or sparing or dependent or receptor* or sensitivity or contracept*)) or anti-glucocortic* or anabol*)) |
